# Sex differences in the management and outcome of acute coronary syndrome—Still an issue of equal treatment?

**DOI:** 10.1007/s00508-023-02302-4

**Published:** 2023-11-23

**Authors:** Roya Anahita Mousavi, Gudrun Lamm, Maximilian Will, Konstantin Schwarz, Julia Mascherbauer

**Affiliations:** 1 Department of Internal Medicine 3/Cardiology, University Hospital St. Pölten, St. Pölten, Austria; 2 Karl Landsteiner University of Health Sciences, Krems, Austria; 3 Medical University of Vienna, Vienna, Austria

**Keywords:** Gender differences, Coronary artery disease, Treatment, Mortality, Myocardial infarction

## Abstract

Significant sex-specific differences were described in the presentation, management and outcome of acute coronary syndrom (ACS) patients. Female ACS patients more often presented with noncardiac symptoms, which lead to significant time delays between symptom onset and treatment. Furthermore, multiple studies from various countries described that women with ACS were less likely to receive the medical or reperfusion therapy recommended by the respective guidelines, resulting in higher in-hospital mortality rates.

The treating physicians and the patients need to be more aware of the described differences to ensure the best possible medical care for ACS patients, irrespective of sex.

## Introduction

Acute coronary syndrome (ACS) represents an urgent/emergent condition that is associated with significant mortality. Sex-related differences in presentation, management and outcome of ACS have repeatedly been postulated. Very recently, a Portuguese research group reported that in a large contemporary cohort women had a significantly higher risk of cardiovascular events and death after ST-elevation myocardial infarction (STEMI) submitted to percutaneous coronary intervention (PCI), even after multivariable correction for confounders [1]. Such disparities, if they do exist, would be indeed troubling and would urgently require efforts to address them.

This review aimed to summarize the existing literature and current knowledge regarding sex-specific differences in patients presenting with ACS.

## Sex differences in ACS presentation and management

For long it has been postulated that women with ACS frequently present with “atypical” or noncardiac symptoms, such as nausea, vomiting, neck and back pain [2–4]. Recently, the terms “typical and atypical” were critically discussed because of the lack of a reference group for “typical” chest pain [5]. As a result, the 2021 guidelines of the American College of Cardiology for the management of chest pain stated that the terms “cardiac or noncardiac” symptoms should be used instead of “typical or atypical” [6]. Chest pain has been shown to represent the main ACS symptom in both sexes [7], and “typical” or “cardiac” symptoms were reported to have a greater predictive value in women than in men [8].

There are consistent data that women presenting with ACS are significantly older and suffer from more comorbidities than males [1, 9–12]. In addition, the time delay between symptom onset and treatment of ACS has been shown to be significantly longer in women. A study from Vienna on 4593 patients reported significant delays in onset of pain to first medical contact in female STEMI patients [13]. Similarly, Stehli et al. observed significant time delays between symptom onset and presentation in women with STEMI, even after adjustment for age and comorbidities [14]. A large study from China, including over 82,000 patients, confirmed delays in the presentation of female STEMI patients, who presented 1.4 h later than men [9]. Marinho et al. reported a longer door-to-balloon time in female ACS patients younger than 55 years as compared to men [1] and another study from Australia on 4859 ACS patients observed delayed arrivals of female patients in the emergency department [15]. Finally, a recent review including 43 studies stated that in over 90% of them longer symptom-to-balloon or door-to-balloon times in female STEMI patients as compared to men were reported [16]. As an explanation most authors discussed that women more often presented with noncardiac symptoms, leading to misinterpretation due to a lack of awareness of both patients and doctors [9, 14, 16]. Furthermore, the culprit lesion was more frequently reported to be the right or circumflex coronary artery in women while men more often presented with left anterior descending artery lesions, which lead to more subtle electrocardiogram changes in women [14].

## Sex differences in ACS treatment

In addition to the reports on sex discrepancies in presentation and acute management of ACS, disparities also exist with respect to treatment. Women were less likely to receive evidence-based ACS treatment including early dual antiplatelet therapy, heparins, reperfusion therapy and secondary prevention treatment in a large Chinese study [9]. Similar observations were reported from Germany, where women were significantly less likely to undergo PCI and coronary artery bypass graft surgery (CABG) than men [17]. In line, another large study from Switzerland including > 220,000 ACS patients reported a lower likelihood to receive PCI or CABG for females as compared to males and also found less women to be admitted to the intensive care unit [12]. Late presentation due to noncardiac symptoms, followed by a lower proportion of reperfusion therapy in female patients was discussed. Furthermore, the fact that women were older and suffered from more comorbidities when presenting with ACS may have affected the eligibility for aggressive treatment [9, 17, 18]. However, that such differences were due to older age in women suffering from ACS was denied by a large prospective Swedish registry which found that women with STEMI were less likely to undergo coronary angiography and PCI regardless of age [19]. The authors furthermore found that women were less likely to be prescribed evidence-based treatment after ACS and finally stated that a sexist bias could not be ruled out as their findings were not supported by current guidelines [19]. On the contrary, registry data from Canada showed that the rate of prescribed evidence-based medical treatment was higher in women than in men after hospital discharge [20].

## Sex differences in short-term ACS outcome

Evidence regarding sex-specific differences in outcome following ACS revealed conflicting results. Women with ACS were shown to be at a higher risk of bleeding, related to smaller body volume, older age, and smaller blood vessels [21, 22]. Regarding PCI in STEMI only, data from Australia showed a higher adjusted 30-day mortality rate in women [14]. Furthermore, female sex was identified as an independent predictor for in-hospital mortality in STEMI but not NSTEMI patients in 875,735 German patients [17]. A recent large study from the USA by Ashraf et al. found a higher risk-adjusted in-hospital mortality of female STEMI patients treated with PCI or CABG from 2017–2019 while no sex differences were found in NSTEMI patients treated with PCI in that period [23]. However, when NSTEMI was treated with CAGB, women had a higher mortality risk [23]. A higher adjusted risk of in-hospital mortality in female ACS patients treated with CABG has also been described by several other studies with large sample sizes [24–27]. This finding was discussed as being due to smaller coronary vessels in women, increasing the likelihood of acute bypass graft failure, in addition to longer treatment delays in females with ACS [23, 24].

On the contrary, only two studies reported no significant sex-related differences in short-term survival after ACS, treated with PCI or CABG, after adjustment for clinical characteristics [10, 28].

## Sex differences in long-term ACS outcome

Inconsistent results were reported regarding the impact of sex on long-term ACS mortality. Several large studies found no sex-specific differences after adjusting for clinical risk factors in ACS patients treated with or without PCI or CABG [13, 18, 20, 28, 29], while a more recent study described a lower long-term ACS mortality risk in women as compared to men [30]. Conversely, a very recent single center study from Portugal that retrospectively included 884 STEMI patients, found a significantly higher 5‑year mortality risk in female patients, even after propensity score matching [1].

As for ACS patients treated with CABG a higher all-cause long-term mortality has been observed in male patients [24, 31]. Conversely, another study observed no sex differences in long-term mortality in CABG patients, however, women experienced a higher risk of major adverse cardiac events [32].

## Conclusion

Significant sex-specific differences were described in the presentation, management and outcome of acute coronary syndrome (ACS) patients. Female ACS patients more often presented with noncardiac symptoms, which lead to significant time delays between symptom onset and treatment. Furthermore, multiple studies from various countries described that women with ACS were less likely to receive the medical or reperfusion therapy recommended by the respective guidelines, resulting in higher in-hospital mortality rates.

The treating physicians and the patients need to be more aware of the described differences to ensure the best possible medical care for ACS patients, irrespective of sex.
